# Surface termination effects on Raman spectra of Ti_3_C_2_T_*x*_ MXenes: an *in situ* UHV analysis

**DOI:** 10.1039/d4cp02197e

**Published:** 2024-07-24

**Authors:** Julian Plaickner, Tristan Petit, Peer Bärmann, Thorsten Schultz, Norbert Koch, Norbert Esser

**Affiliations:** a Technische Universität Berlin, Institut für Festkörperphysik Hardenbergstraße 36 10623 Berlin Germany julian.plaickner@tu-berlin.de; b Helmholtz-Zentrum Berlin für Materialien und Energie GmbH Albert-Einstein-Straße 15 12489 Berlin Germany; c Leibniz-Institut für Analytische Wissenschaften-ISAS-e.V. Schwarzschildstraße 8 12489 Berlin Germany; d Institut für Physik & IRIS Adlershof, Humboldt-Universität zu Berlin 12489 Berlin Germany

## Abstract

Ti_3_C_2_T_*x*_ MXenes have typically a mixed surface termination of oxygen, hydroxyl and fluorine groups (T_*x*_). In this work, we investigate the influence of the surface termination on the vibrational properties of Ti_3_C_2_T_*x*_ by performing thermal desorption and *in situ* Raman spectroscopy in ultra-high-vacuum (UHV). Significant changes in the Raman spectra occur after annealing above 600 °C, correlated with the desorption of approximately 80% of the fluorine termination, as confirmed by mass spectrometry and X-ray photoemission spectra. In particular, the intense Raman mode at 203 cm^−1^, usually attributed to a Ti–C-layer stretching vibration, is strongly damped upon fluorine desorption, while the broad spectral features between 220 and 680 cm^−1^, usually attributed to surface group vibrations, are not changing significantly. We show that the Raman spectra and the change induced by fluorine desorption are well represented by the phonon density of states instead of zone-center phonon modes. Disorder-induced Raman scattering strongly contributes to the Raman spectra. Moreover, due to the metallic nature of MXenes, charge density fluctuation scattering contributes as well. We show that the two scattering mechanisms, deformation potential and charge density fluctuation, may lead to opposite interpretations concerning the symmetry of the fluorine-related mode at 203 cm^−1^. This study provides new insights into the interpretation of the Raman spectra of MXenes, especially regarding the relation between surface chemistry and vibrational spectroscopy.

## Abstract 2

This work provides new insights into the interpretation of Raman spectra of Ti_3_C_2_T_*x*_ MXenes by thermal desorption of surface terminations in vacuum. Despite broad spectral features, the contribution of the fluorine termination is clearly identified.

## Graphical abstract

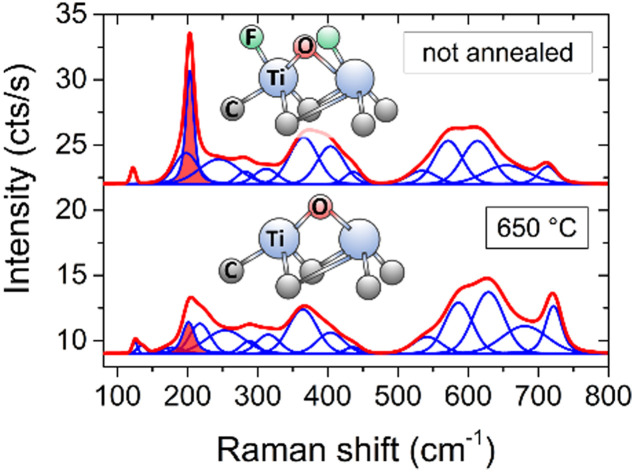

## Introduction

MXenes, a family of 2D transition metal carbides/nitrides, are highly attractive for a multitude of applications, *e.g.* in electrochemical energy storage,^1–3^ gas sensors,^4,5^ photodetection,^6^ biomedical applications,^7,8^ and water desalination.^9,10^ They are synthesized by etching of the A element from M_*n*+1_AX_*n*_ precursors (M = transition metal, *n* = 1, 2, 3, A = group III or IV element, X = C or N), so-called MAX phases, resulting in 2D M_*n*+1_X_*n*_T_*x*_ MXenes (where T_*x*_ is the surface termination group).^11–13^ The most studied MXene so far, Ti_3_C_2_T_*x*_ MXene, is produced in particular by etching a Ti_3_AlC_2_ MAX phase. As a result of the chemical exfoliation and etching process in HF:HCl etchant, the obtained Ti_3_C_2_T_*x*_ MXene has a mixed termination of T_*x*_ = OH, O and/or F.^1,14,15^ Ti_3_C_2_T_*x*_ is metallic, and the surface termination T_*x*_ has been shown to have a significant influence on fundamental material properties such as electronic band structure, band occupation, Fermi surface, and work function.^16,17^ Desorption of the surface termination may lead, moreover, to highly reactive surfaces. A comprehensive knowledge of the surface chemistry of MXenes is therefore critical for understanding their physical and chemical properties that will affect technological applications.^18–20^

The surface chemistry of Ti_3_C_2_T_*x*_ MXenes has been probed with X-ray photoelectron spectroscopy (XPS)^14,21,22^ and other experimental techniques, such as Raman spectroscopy.^23,24^ IR spectroscopy was shown to be mostly sensitive to intercalated species rather than to MXene surface chemistry.^25^ The surface termination of Ti_3_C_2_T_*x*_ MXenes was found to be composed of an inhomogeneous mixture of F-, O- and OH-groups.^21,22,26^

By *ab initio* theory, atomic geometries and related vibrational modes have been calculated for unterminated and purely F-, O- or OH-terminated MXene model structures.^27^ More recently, a combined *ab initio*-/force-field dynamics simulation of Raman spectra for different amounts of O- and OH-groups at the surface including structural disorder was carried out.^28^ It was shown that both effects, surface termination and structural disorder, have a very large impact on the vibrational spectra, which makes the assignment of the vibrational signatures difficult.

In this work, we characterize the vibrational properties of Ti_3_C_2_T_*x*_ MXene for the first time under chemically inert conditions in ultra-high vacuum (UHV). In UHV, the surface termination of MXenes may be modified by thermal desorption while a progressive oxidation of the MXene is avoided. *In situ* Raman spectroscopy on MXenes in UHV gives clear evidence of structural changes upon desorption of fluorine surface groups up to a temperature of 675 °C.

## Experimental details

### Ti_3_C_2_T_*x*_ MXene synthesis and film preparation

The Al-rich Ti_3_AlC_2_ precursor with a TiC : Ti : Al ratio of 2 : 1.25 : 2.2 was mixed with 20 ml of etchant (a 6 : 3 : 1 mixture of HCl, H_2_O, HF) and stirred at 400 rpm for 24 hours at 35 °C. The etched Al–Ti_3_C_2_ was washed with distilled H_2_O *via* centrifugation and decantation cycles until the supernatant reached a pH value of 6. Then it was dispersed in a 0.5 M solution of LiCl to start the delamination process by stirring at 400 rpm for a minimum of 4 hours. The MXene/LiCl suspension is again washed *via* centrifugation and decantation cycles. More details about the Ti_3_C_2_T_*x*_ MXene synthesis can be found elsewhere.^29,30^ Finally, a thick Ti_3_C_2_T_*x*_ MXene film composed of overlapping few-layered flakes were obtained by drop-casting the colloidal dispersion on a Si substrate (with native oxide), similarly as in ref. 25.

### Ultra-high vacuum (UHV) setup

The MXene sample was drop casted and transferred into the UHV chamber with a base pressure of 10^−10^ mbar. Sample heating was realized by heating a ceramic-isolated tungsten filament placed on the back of the sample holder. The sample temperature was measured by using an infrared pyrometer focused on the area of the Si substrate not covered by the MXene flakes (distributed only at the center of the substrate). Mass spectra of the residual gas in the vacuum chamber and of the desorbed species during the annealing cycles were acquired by using a quadrupole mass spectrometer. Annealing of the MXene sample increased the pressure in the UHV chamber in the mid 10^−9^ mbar range. The annealing sequence of the sample was realized as follows: the sample was kept at a fixed temperature for a variable time (usually between 1 and 2 hours) until the pressure in the UHV chamber decreased back to the 10^−10^ mbar range. Then, after slowly cooling down to room temperature, Raman spectra were acquired.

### Raman spectroscopy

Raman spectra were recorded *in situ* in UHV in near-backscattering geometry (60° between incident and scattered beam, which was normal to the sample surface).^31^ A solid-state laser at 660 nm with power of 300 mW was used for Raman excitation. The laser spot on the sample surface was focused to an area of approximately 100 μm^2^. Fresnel rhombs in the incident and scattered light beam were used for the polarization adjustment of light. Since no polarization analyzer was in the Raman scattered beam, the degree of linear polarization was only approximately 85%, as defined by the monochromator transmission function. The Raman spectrometer is a subtractive triple grating spectrograph with a Si CCD detector. A detailed description of the *in situ* UHV Raman setup can be found elsewhere.^32^ The collected Raman spectra were calibrated by using the spectral lines of a Hg–Ar lamp. The full width at half maximum of the measured lines was taken as spectral resolution and found equal to 2 cm^−1^ for the 660 nm laser excitation at 100 μm slit width.

### X-ray photoelectron spectroscopy (XPS)

XPS measurements were conducted using a JEOL JPS-9030 setup with a base pressure of 10^−9^ mbar, employing a monochromatic Al source with an angle of incidence of 10° with respect to the sample surface and a power of 300 W for excitation. A hemispherical analyser with pass energies of 50 eV (surveys) and 10 eV (narrow scans) was used to detect the emitted photoelectrons under an angle of 10° off from normal emission. The binding energy scale was calibrated by measuring sputter-cleaned gold and copper foils and setting the Au 4f_7/2_ peak to 84.0 eV and the Cu 2p_3/2_ peak to 932.6 eV. No signs of charging were observed during the measurements. All samples were exposed to air for about the same time before introduction into the UHV system.

## Results and discussion

### Vibrational and electronic Raman scattering at MXenes

**Fig. 1:**
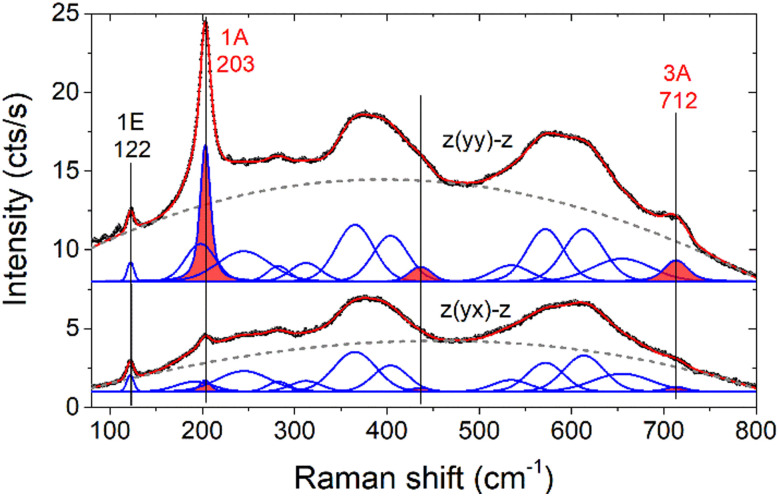
Raman spectra of Ti_3_C_2_T_*x*_ MXene flakes in parallel (top) and crossed (bottom) polarization configuration. Experiment (black dots) and the resulting fit (red, solid line) are indicated together with the individual fitting components (blue, solid line) and the background (grey, dashed line). The observed bands with A-symmetry are filled with red color.

Raman spectra of Ti_3_C_2_T_*x*_ MXene flakes after transfer into UHV are shown in Fig. 1, measured in parallel and crossed polarization. Polarization selection rules are expressed in the Porto notation^31^ (*z*(*yy*)–*z* and *z*(*yx*)–*z*), where *x* and *y* are two perpendicular directions in the surface plane, while *z* is the direction perpendicular to the surface. The measured Raman spectra were modelled by a combination of a cubic function (dashed grey line) for the background and several Voigt functions for the superimposed bands. These bands have been attributed to vibrational Raman scattering^23,24^ and are modelled in a similar way to Sarycheva *et al.*^23^

In the following, we discuss the different contributions. First, we attribute the broad background in the spectrum to electronic Raman scattering (ERS), *i.e.* inelastic light scattering by conduction band electrons in the vicinity of the Fermi surface level.^16^ ERS is typical for metals and has been reported, for instance, on d-band metals.^33,34^

Second, the phonon bands at 203, 436 and 712 cm^−1^ (marked with red color), visible predominantly in parallel polarization configuration, should refer to A-modes, while the sharp band at 122 cm^−1^ and several spectrally broad bands between 220 and 680 cm^−1^, visible in both parallel and crossed polarization configurations, to E-modes. We would like to note that the mode symmetry assignment is strictly valid only for deformation potential scattering of modes at the Brillouin zone center.^35^ According to the layered trigonal structure of MXene (point group *D*_3d_), A- and E-modes with out-of-plane and in-plane atomic displacements exist, respectively. The vibrational eigenmodes, phonon dispersion and phonon density of states of MXene model structures have been calculated by *ab initio* theory.^27^ At the Brillouin zone center of unterminated MXene, low-frequency modes at 158 (E_g_) and 228 cm^−1^ (A_1g_) refer to the in-plane and out-of-plane vibrations of the upper and lower Ti–C-layers, whereas high-frequency modes at 621 (E_g_) and 599 cm^−1^ (A_1g_) refer to the in-plane- and out-of-plane-vibration of the central C-layers of the MXene structure. With F-, O-, or OH-termination, additional modes arise, most of which are strongly coupled to the Ti–C-layer vibrations. However, the calculated zone-center eigenmodes do not well reflect the experimental Raman spectrum. The effect of a disordered arrangement of different surface termination groups was recently shown to reproduce much better Raman spectra of Ti_3_C_2_T_*x*_ MXenes, using a force-field dynamics calculation for mixed O- and OH-surface terminations.^28^ Accordingly, the narrow lines at 122 (E), 203 (A), and 712 cm^−1^ (A) should be mostly related to zone center modes of MXene, while the broad spectral features between 220 and 680 cm^−1^ should refer to a weighted average of phonons over the entire Brillouin zone. The force-field dynamics calculation for mixed O- and OH-termination demonstrates that the Raman spectra depend both on the relative amount and local distribution of the surface termination species.^28^

Overall, the Raman spectra of the as-prepared Ti_3_C_2_T_*x*_ MXenes in UHV are characteristic of a disordered compound metal. The large electron density at the Fermi level^16^ leads to a broad, unstructured spectrum of electronic Raman scattering from intraband excitations, superimposed by phonon bands. The disorder and the mixed surface termination leads to broadening of the phonon bands. Vibrational Raman spectra refer to the superposition of the density of phonon states and phonon modes at the Brillouin zone center. Three narrow Raman bands arise at 122 cm^−1^, 203 cm^−1^, and 712 cm^−1^ should be related to in-plane and out-of-plane vibrations of Ti–C and C atomic layers, according to previous work.^23,27^

### Annealing of Ti_3_C_2_T_*x*_ MXenes in UHV

**Fig. 2:**
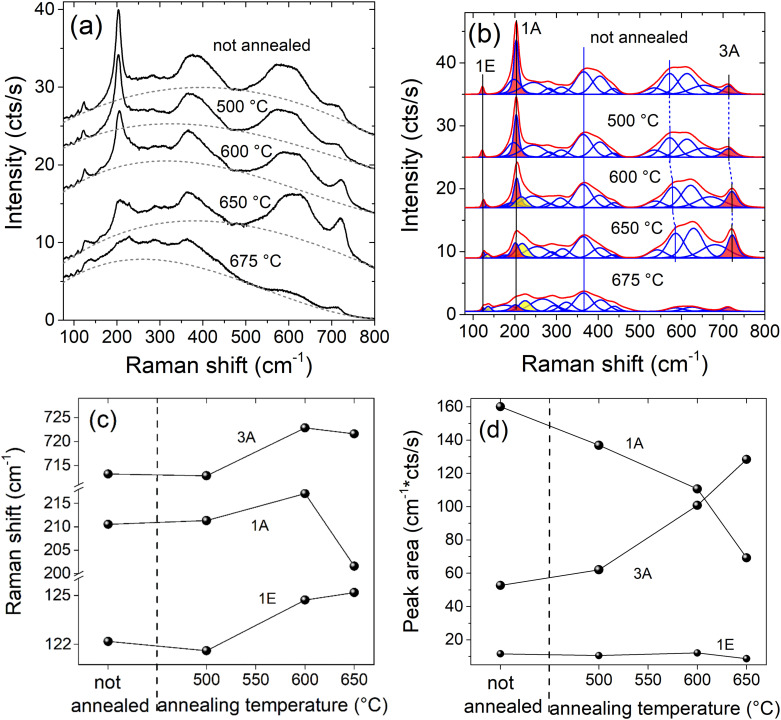
Evolution of Raman spectra upon annealing the Ti_3_C_2_T_*x*_ MXene sample at different temperatures; (a) experimental spectra (black) and cubic background function (grey, dashed); (b) individual fitting components (blue) and total spectrum after background subtraction (red); (c) Raman shifts and (d) intensities of the relevant bands filled with red color in (b).

The Raman spectra measured at room temperature after annealing in UHV are shown in Fig. 2(a). After subtraction of the ERS background, the spectra and the modelled phonon bands are shown in Fig. 2(b). After fitting the spectrum of the non-annealed sample, the obtained set of functions is used as starting configuration for fitting the spectrum after the first annealing step (and so on for the successive annealing steps). After annealing up to 500 °C, the phonon spectrum and the background do not change significantly. However, evident changes are found in both components for higher annealing temperatures. For annealing at 600 and 650 °C, the low-frequency E- and A-modes (Ti–C layer) reduce in intensity and broaden significantly, while the high frequency A-mode (C-layer) sharpens and increases in intensity. Moreover, new bands on the high-energy shoulders of the Ti–C–E and -A-modes appear, *i.e.* at 134 and 217 cm^−1^ (filled with yellow color). The broad disorder-related bands in between change only slightly: the lower frequency bands between 350 and 450 cm^−1^ decrease in intensity, while the higher frequency bands between 500 and 750 cm^−1^ increase in intensity and shift to higher frequency upon annealing. After annealing at 675 °C, both the ERS component and the phonon component change significantly. The ERS background shifts to low frequency while all the phonon bands at frequencies above 500 cm^−1^ show a dramatic reduction in intensity. For annealing below 675 °C, Fig. 2(b) reveals that the intensity of the three narrow E- and A-Raman bands (filled in red) are particularly affected. The Raman shift and intensity of the three narrow E- and A-Raman bands as function of the annealing temperature are shown in Fig. 2(c) and (d). The bands 1A (Ti–C stretch) and 3A (C-layer) show opposite trends concerning both frequency shift and intensity variation. The band 1E (Ti–C bending) shows an increase in frequency, but it preserves its intensity. We would like to note that the change in in mode intensities, Fig. 2(d), could be partly related also to Raman resonance effects,^35^*i.e.* changes of the electronic band structure upon annealing. This holds also for the C-layer related broad vibrational band above 500 cm^−1^.

Because ERS and phonon components show only minor changes for annealing up to 500 °C, we conclude that the MXene sheets maintain their atomic and electronic structure up to this temperature. However, at 600 °C new bands appear at 134 and 217 cm^−1^ (filled with yellow color) in the Raman spectra. We would like to note that these bands cannot be related to MXene oxidation,^23^ because the samples are held in UHV. Also in our spectra there is no indication of any carbon-related bands between 1300 and 1600 cm^−1^ up to the maximum annealing temperature of 675 °C (spectral range not shown here). Therefore, we conclude that the intensity reduction of the low frequency E- and A-mode together with the appearance of weak features at 134 and 217 cm^−1^ and the change in line shape of the broad bands at 350 to 450 cm^−1^ and at 500 to 750 cm^−1^ are related to the change in surface termination.

**Fig. 3:**
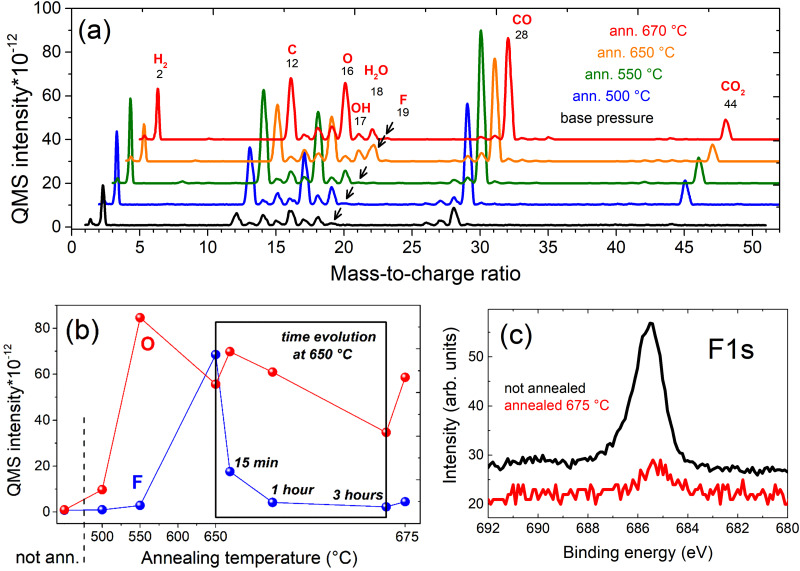
(a) Mass spectra acquired during annealing of the Ti_3_C_2_T_*x*_ MXene sample at different temperatures (spectra are normalized to the peak of N (14) for clarity). (b) QMS intensities of O and F as function of temperature. The inset shows the time dependence of the signals at 650 °C. (c) F 1s XPS spectrum before and after annealing at 675 °C.

To identify the desorbed species from the MXene sample, mass spectra of the residual gas in the vacuum chamber were acquired before and during the annealing process, as shown in Fig. 3(a). The interpretation of the spectra is not straightforward, because not only the sample but also parts of the sample holder and the heating filament itself are degassing. This explains the significant increase of C and CO during annealing. The evolution of the mass spectrum signals of the two surface termination species of the Ti_3_C_2_T_*x*_ MXene surface, O and F, as function of temperature is shown in Fig. 3(b). The inset shows the time evolution at fixed annealing temperature of 650 °C. In contrast to O, F shows an exponential decay in time at a fixed annealing temperature of 650 °C, which is typical for a desorption process. In agreement, the F 1s XPS spectra in Fig. 3(c), acquired before and after annealing at 675 °C, indicate a significant reduction of the fluorine concentration from 17% to 3%. Accordingly, XPS studies showed the desorption of 60–80% of fluorine above 500 °C annealing temperature while the oxygen concentration at the surface remained unaffected.^26^

**Fig. 4:**
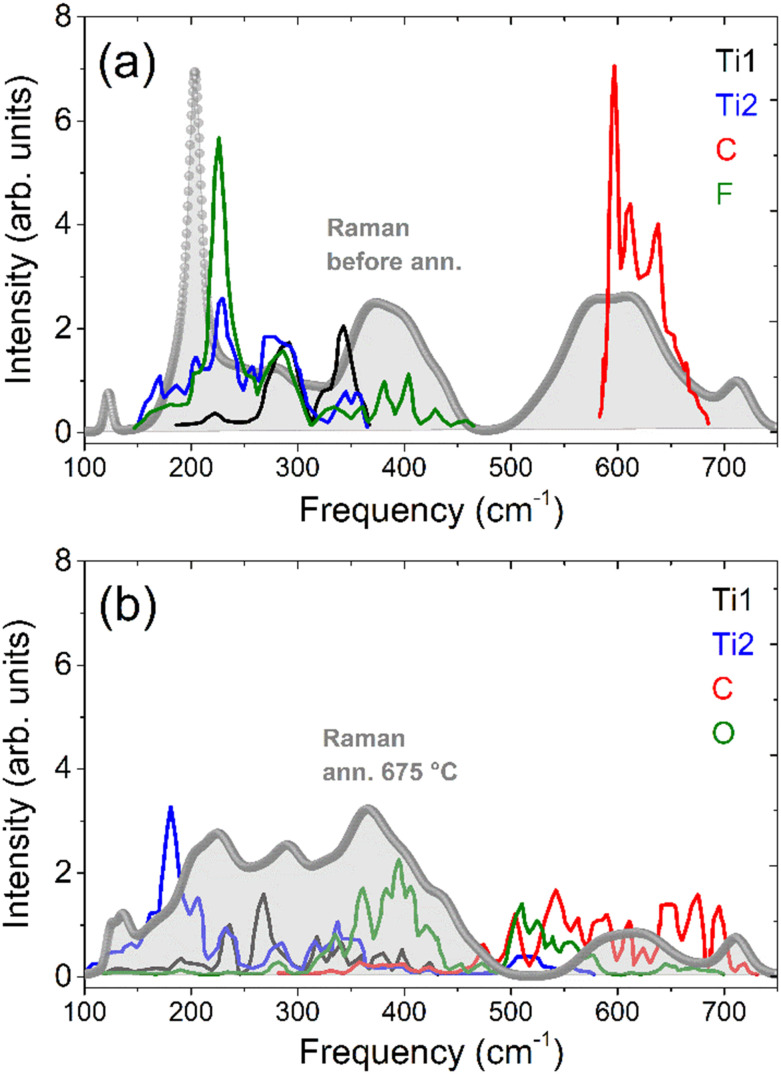
Comparison of the Raman spectra before (a) and after (b) fluorine desorption with the calculated phonon density of states for a purely terminated F- and O-model sheet from Hu *et al.*^27^

The experimentally observed changes in the Raman spectra can be explained by considering the eigenmodes and the partial phonon density of states (DOS) calculated by Hu *et al.*^27^ In the following, we limit the discussion to F- and O-terminated Mxene model structure since recent XPS work an analogously prepared samples did not show any evidence for OH-termination in UHV^21,22,26^ (this may differ in humid environments). A reproduction of the calculated phonon DOS from Hu *et al.*^27^ for F- and O-terminated Ti_3_C_2_T_*x*_ model sheets is shown in Fig. 4, superimposed with the Raman spectra before and after the annealing procedure. The vibrational density of an F-terminated model sheet is characterized by a strong peak around 200 cm^−1^, associated with a mixed F–Ti-vibration mode with large F-contribution. This feature fits well with the 1A Raman band at 203 cm^−1^ before annealing. In contrast, the O-terminated model sheet has in general a much broader distribution of spectral density over the entire range and a purely Ti vibration related mode left at 200 cm^−1^ with a lower density. Above 550 cm^−1^, a C-layer related branch arises on both F- and O-terminated MXene, but for O-termination showing a much broader and lower spectral distribution. This is reflected rather well in the Raman spectrum, showing a significant damping of the spectral features above 500 cm^−1^ after annealing. One should keep in mind that the real structure is more complex than that model structure, as the initial state is a mixed F- and O-surface termination and possible changes in the adsorption sites of the surface are not included in the calculation. Moreover, changes of the Raman scattering intensity as possibly related to resonance effects are not included as well. Nevertheless, the comparison of the phonon DOS with the Raman spectra shows clearly the effect of fluorine desorption.

We would like to note that in the calculated phonon DOS for the F-termination, the sharp maximum at 220 cm^−1^ arises from the dispersion of the fluorine-related E-symmetry vibration at 231 cm^−1^ in the Brillouin zone center, superimposed with other vibrational bands at higher *k*-values in the Brillouin zone.^27^ This assignment seems to conflict with the Raman polarization selection rules that show a mode of A-symmetry at 203 cm^−1^ (see Fig. 1).

However, it has to be considered that the polarization selection rules according to the point group symmetry refer to the deformation potential scattering of zone center phonon modes.^35^ Therefore, the preferential parallel polarization may be due to disorder-induced Raman scattering. Moreover, in the case of metallic materials, inelastic light scattering by charge density fluctuations may be the dominant scattering mechanism instead of deformation potential scattering. This scattering mechanism gives rise to polarized light scattering in parallel polarization configuration.^36,37^ Therefore, the strong preference for polarized scattering may be an indication of disorder or charge density scattering of the E-symmetry mode and consequently the correct assignment of the 203 cm^−1^ mode may be the E-type shear vibration localized in the uppermost fluorine layer.

Finally, a strong impact of the surface termination on the vibrational and electronic response of the MXene sheet is shown, but there is no evidence of large mode frequency shifts upon fluorine depletion, as suggested from the model sheet calculations. This is certainly related to the effect of surface (and possibly bulk) disorder. Nevertheless, the Raman signature depends uniquely on the surface structure and can be used as a fingerprint of structural and electronic properties. Annealing of the as-prepared MXene in UHV induces F-depletion and restructuring of the surface with O-termination. Most characteristic for the fluorine depletion is the strong attenuation of the F mode at 203 cm^−1^. Other distinct surface vibrational modes of the T_*x*_-groups are not evident, due to the strong coupling between surface termination groups and underlying Ti–C-layers. Thus, the Ti–C layer vibrational bands change also strongly upon F-depletion.

## Conclusions

The effect of annealing on the Ti_3_C_2_T_*x*_ MXene surface structure in UHV has been investigated with Raman spectroscopy. The metallic nature of Ti_3_C_2_T_*x*_ MXene leads to a broad background due to electronic Raman scattering that is superimposed to the phonon bands. Despite disorder-related broadening of the Raman bands, the change of the Raman spectra upon annealing can be interpreted with the support of calculations of the vibrational density of states of MXene model sheets. At annealing above 650 °C, evident changes in the Raman spectra correlate with a fluorine depletion and a persistent oxygen termination. We found that the sharp mode around 200 cm^−1^ is mainly related to the fluorine termination, in contrast to previous literature attributing it to vibrations of the Ti–C layer.

## Data availability

The data of our work are available from the authors upon reasonable request.

## Conflicts of interest

The authors declare no conflict of interests. All co-authors have seen and agree with the contents of the manuscript and there is no financial interest to report. We certify that the submission is original work.
